# Meleney’s gangrene managed with a single extensive debridement and resultant defect closure with abdominoplasty technique – a case report

**DOI:** 10.1097/MS9.0000000000001727

**Published:** 2024-01-15

**Authors:** Somi Ahmed, Niran Maharjan, Niroj Hirachan

**Affiliations:** a Critical Care Unit, Sumeru City Hospital; b Department of Plastic Surgery, Sumeru City Hospital; c Department of Anesthesia, Patan Academy of Health and Science, Lalitpur, Nepal

**Keywords:** abdominoplasty, Meleney's gangrene, case report, radical debridement

## Abstract

**Introduction:**

Meleney’s gangrene, or progressive bacterial synergistic gangrene, is a life-threatening subcutaneous tissue infection and skin necrosis of the abdomen that is persistent and quickly progressing and has documented cultural characteristics of a symbiotic organism. The nobility of this case lies in the use of the modern technique, abdominoplasty, used to close the wound post-radical debridement for Meleney’s gangrene. This uncommon illness has a high fatality rate and requires immediate diagnosis, aggressive antibiotic treatment, and extensive debridement.

**Case presentation:**

We report the case of a 55-year-old female with no known comorbidities, who presented to our center with features of Meleney’s gangrene and pleural effusion. Radical debridement was performed and empirical intravenous antibiotics were administered. The wound was closed using the abdominoplasty approach.

**Clinical discussion:**

Meleney’s gangrene should be identified quickly and treated with wide-spectrum antibiotics and rigorous surgical debridement. It is difficult to diagnose the illness early, and skepticism is strong during this process. An increased risk of death may follow a postponed diagnosis of Meleney’s gangrene. A long-term hospital stay can result from extensive debridement. Furthermore, skin transplants may be required to close wounds in certain instances.

**Conclusion:**

This case is presented to show how early intervention and radical debridement can improve the outcome in cases of Meleney’s gangrene, which is rare and clinically significant. Additionally, this suggests that a cosmetic procedure known as abdominoplasty could be a viable option for wound closure.

## Introduction

Highlights

- Abdominoplasty, also known as a tummy tuck cosmetically used to tighten muscles in the abdominal wall, is used in our case to close the defect caused by extensive surgical debridement of the Meleney gangrene.
- Meleney’s gangrene, a rare type of necrotizing anterior abdominal wall infection common in an immunocompromised state, is seen in a healthy woman in our case report.
- Early detection and extensive surgical debridement followed by closure with abdominoplasty can serve as a safe and effective alternative to conventional multiple debridement and reconstructive surgeries.
- Timely diagnosis and prompt management help to reduce morbidity in the case of Meleney’s gangrene, while abdominoplasty also aids in reducing the duration of hospital stay.

Meleney’s gangrene is a rapidly progressive destructive infection leading to necrosis of the subcutaneous tissue and skin of the abdomen, which may extend to involve the underlying muscle^1^. Since the 18th century, it has been identified and treated as a life-threatening rare condition under several names, such as Fournier’s gangrene, hospital gangrene, and necrotizing fasciitis^2^. Surgeons Dr Brewer and Dr Meleney of New York first wrote about this progressive gangrene in 1926, and in 1931, they further identified the causative microbiological effectors^3^. Meleney’s gangrene is also referred to as Meleney’s ulcer or postoperative synergistic bacterial gangrene which is caused by the interaction between the non-hemolytic *Streptococcus* and hemolytic *Staphylococcus* ^4^. It is a rare type of necrotizing infection involving the anterior abdominal wall, which typically appears in the second week following surgery or moderate trauma^5^. The mainstay of treatment for Meleney’s gangrene involves vigorous debridement and adequate antibiotic coverage. If not treated appropriately, it may lead to serious complications including mortality. This case has been reported following SCARE (Updating Consensus Surgical Case Report) 2023 guidelines^6^.

## Case presentation

A 55-year-old female presented with swelling in her left lower abdomen for 5 days. She was stung in the left lower quadrant of her abdomen by a hoverfly that appeared innocuous while lounging in an open area on a sunny day. She felt the bite but did not care because, initially, nothing was noticeable. The patient was a chronic smoker with no documented comorbidities. The next day, she noticed a small blister at the stung site, which she ruptured with a toothpick, and left it, reassuring herself that it would resolve. The lesion slowly became painful; therefore, she used a painkiller and was confused with the boil. By the sixth day, she had developed pain and redness in the region associated with fever. She visited a nearby hospital on the periphery. She was referred from there, and then they came to a reputed hospital in the vicinity, where she was admitted with the diagnosis of cellulitis and the administration of intravenous antibiotics and other medications. Despite treatment, there was a gradual worsening of the lesion, so the antibiotic was upgraded, after which there was some improvement in the symptoms and blackening of the lesion. However, during resuscitation, the patient collapsed and was advised to move to the ICU.

**Figure 1:**
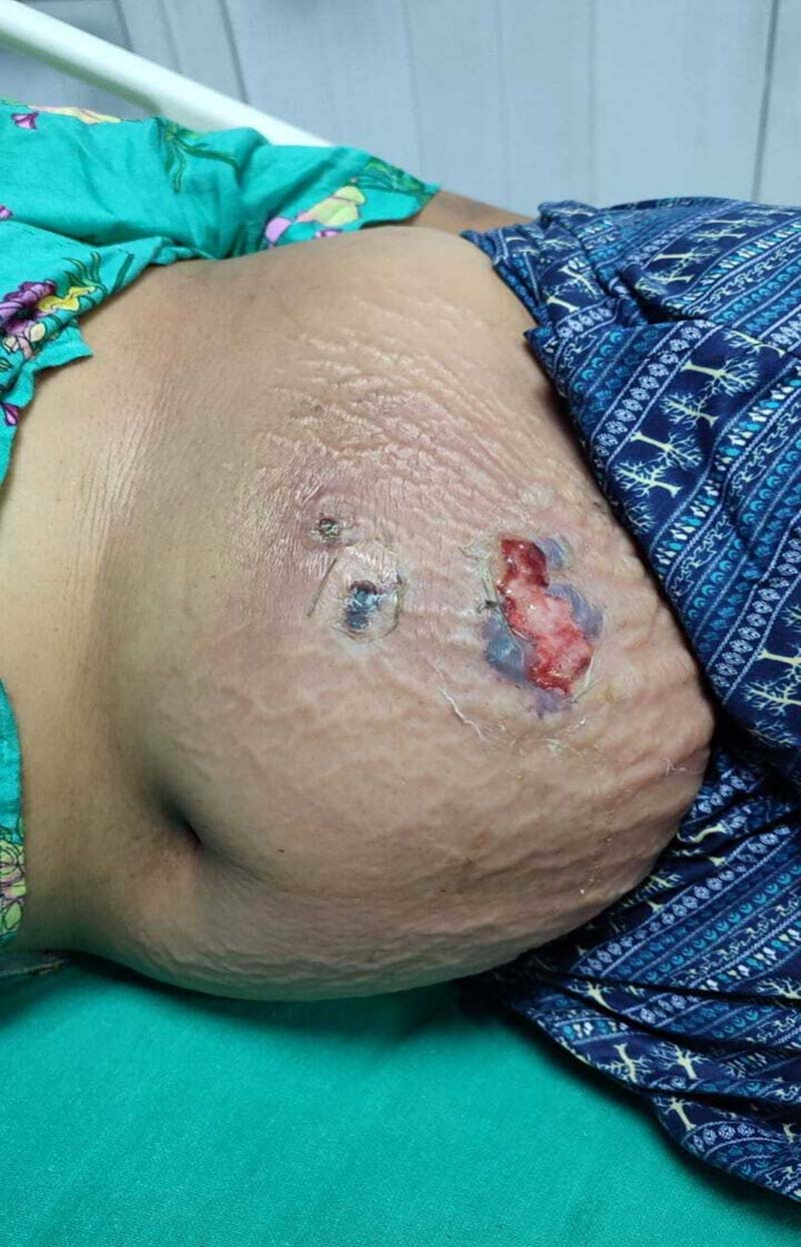
Presentation features: ruptured blisters and necrosed skin.

The patient was shifted to our setup after initial resuscitation in another hospital for 3 days. On arrival, the patient was febrile and had experienced remarkable pain. The patient was hemodynamically and systemically stable. Abdominal examination revealed tender erythematous swelling in the left lower abdomen with blisters, blackening, and peeling of the skin (Fig. 1). The patient was evaluated by a general surgeon and an anesthesiologist and admitted to the ICU with the necessary intravenous support. The next morning, she was consulted for a plastic surgery opinion. Necrotizing fasciitis spreading beyond the discoloration was diagnosed, and exclusive debridement was advised. Owing to the presence of right-sided pleural effusion and worse condition of the chest, along with a total white blood cell count (WCC) of 23 000 cm^3^, she was scheduled to undergo debridement under spinal anesthesia.

**Figure 2:**
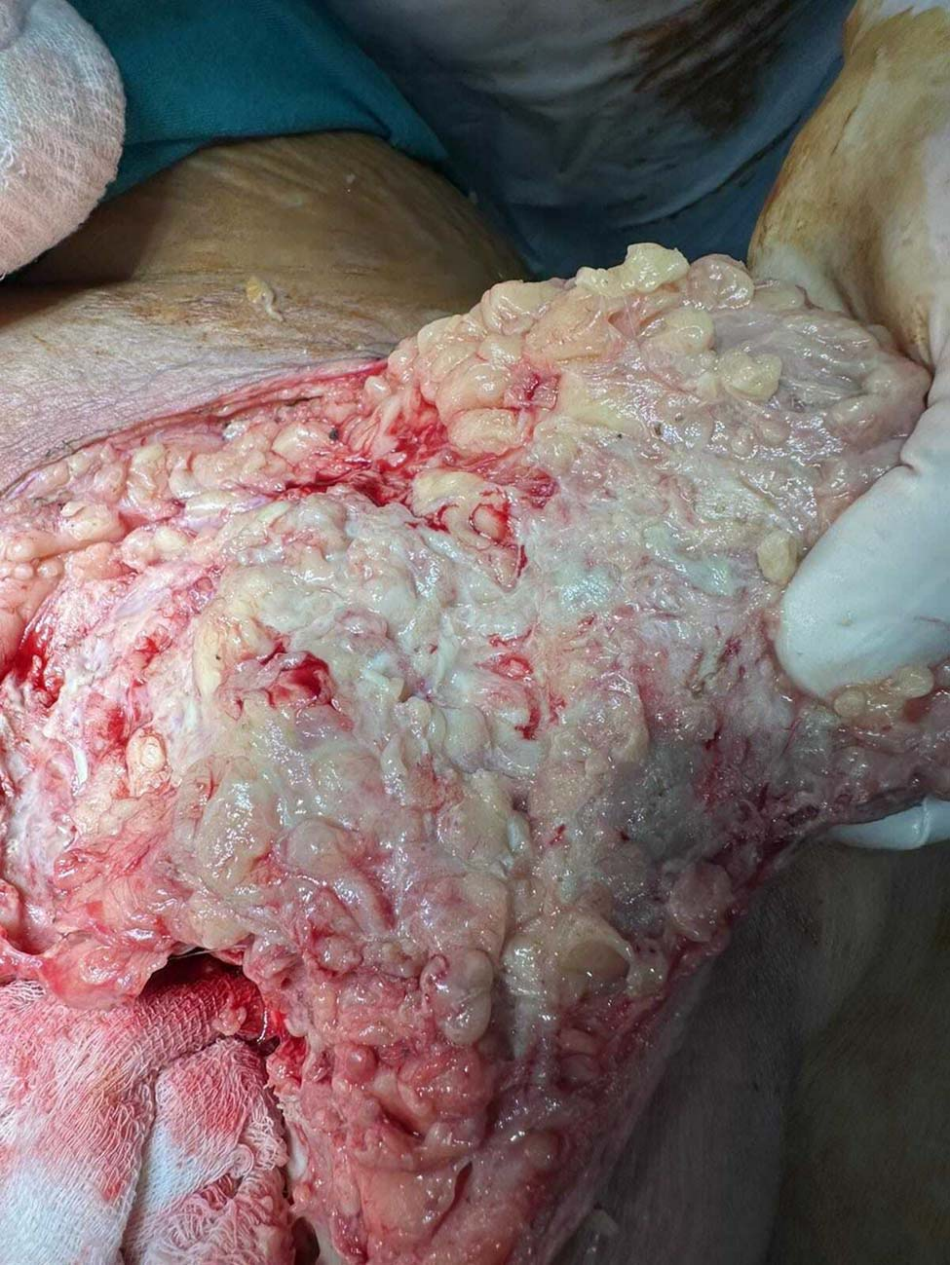
Intraoperative findings of extensive necrotic subcutaneous tissue.

**Figure 3:**
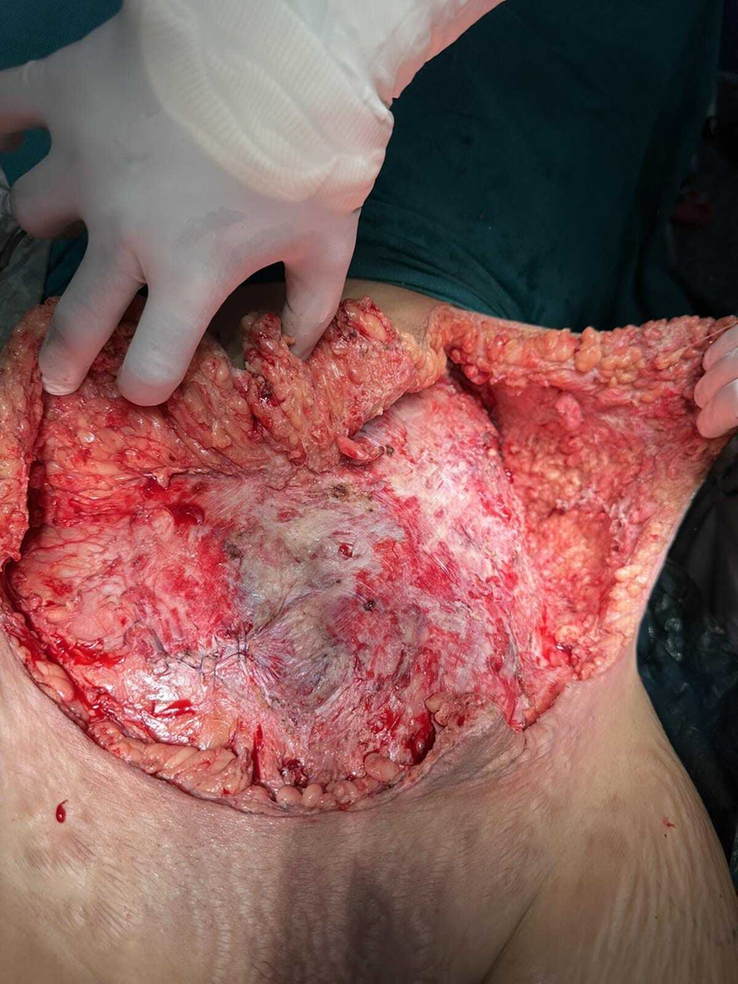
Intraoperative picture of extensive debridement of anterior abdominal wall.

**Figure 4:**
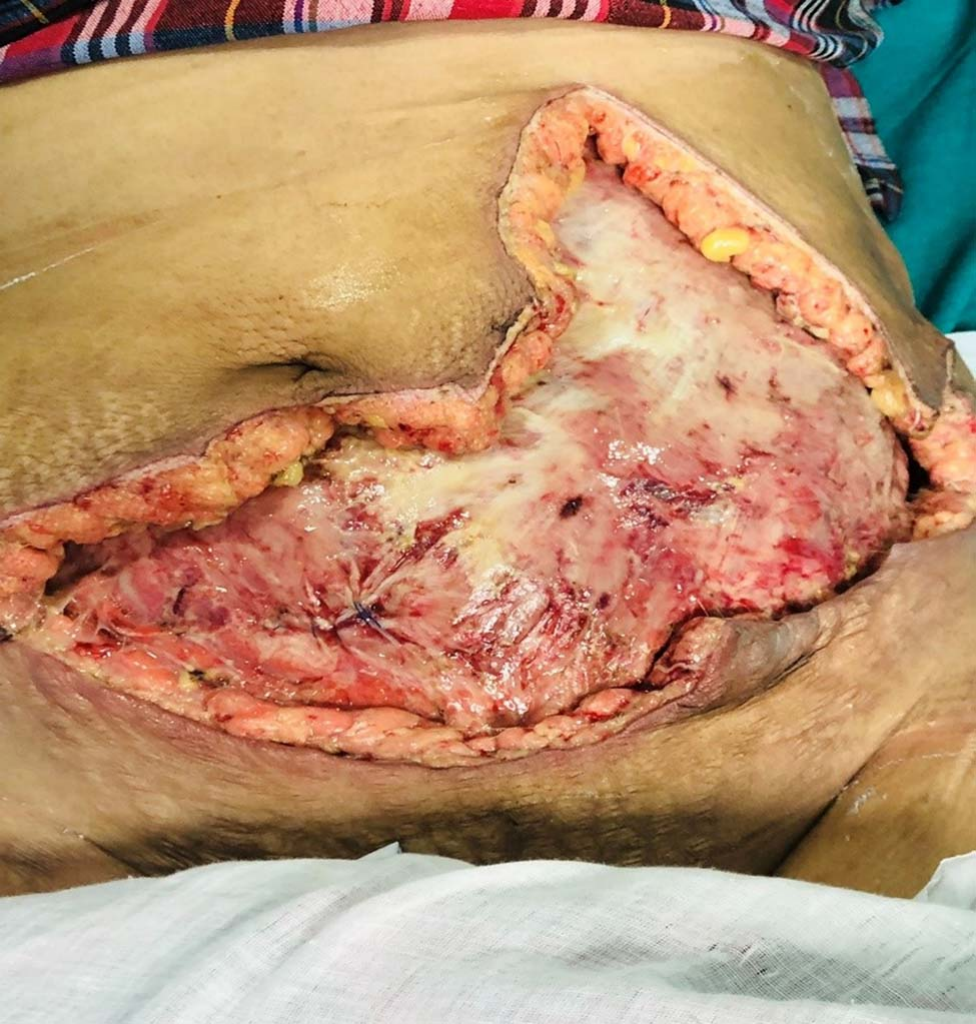
Post-extensive surgical debridement of necrotic tissue.

**Figure 5:**
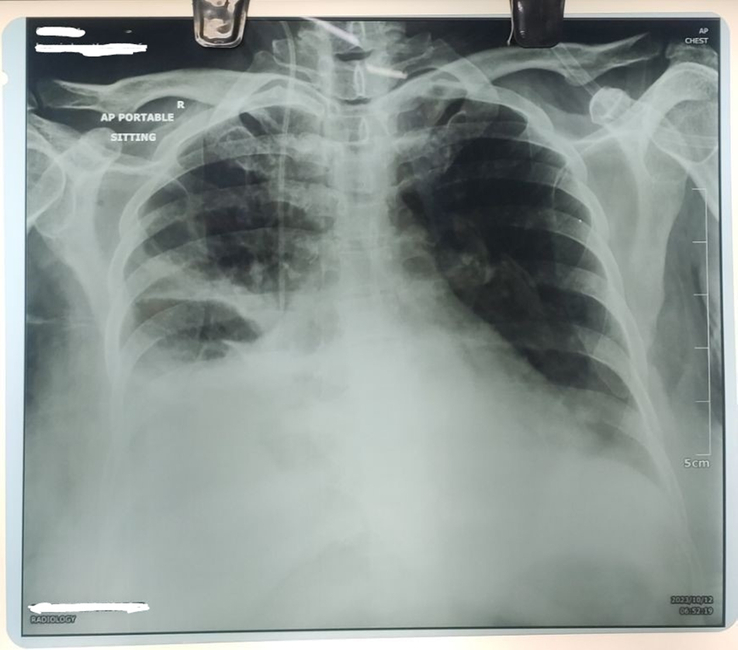
Chest radiograph anteroposterior view showing bilateral pleural effusion prior to pleural tapping.

On the second day of hospital admission, she underwent massive debridement with the removal of all the necrotic and infected fascial layers and subcutaneous tissue (Figs 2, 3). Pre-debridement (Fig. 1) and post-debridement (Fig. 4) pictures depict the level of debridement. The following morning, her condition remained static, with no signs of spreading of the lesion. On the third postoperative day, despite a decrease in the total white blood cell count to 13 300 cm^3^, there was a noticeable increase in the amount of pleural effusion. Diagnostic pleural tapping was performed, and the fluid was sent for investigation. On the fourth postoperative day, the count continued to decline with no obvious evidence of wound spreading; however, an ultrasound of the chest revealed a collection of 550 ml of fluid in the left pleural cavity and 600 ml in the right pleural cavity (Fig. 5). *Staphylococcus aureus* was isolated from the pus culture of the wound sent during debridement, and it was found to be resistant to every antibiotic we employed. Clinical evidence indicated that the prescribed antibiotics were effective; therefore, we substituted culture-resistant levofloxacin with culture-sensitive cloxacillin while continuing with other treatments.

**Figure 6:**
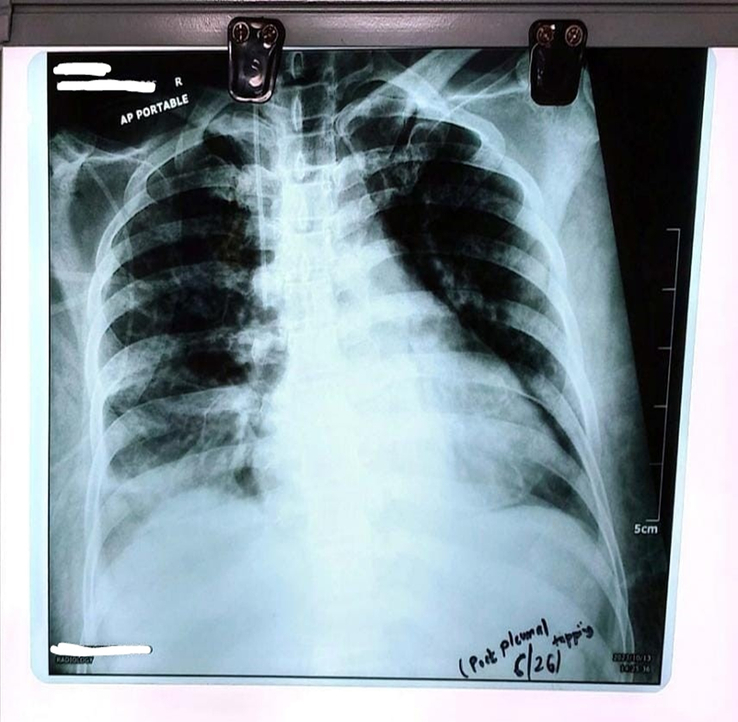
Chest radiograph anteroposterior view post-pleural tapping.

**Figure 7:**
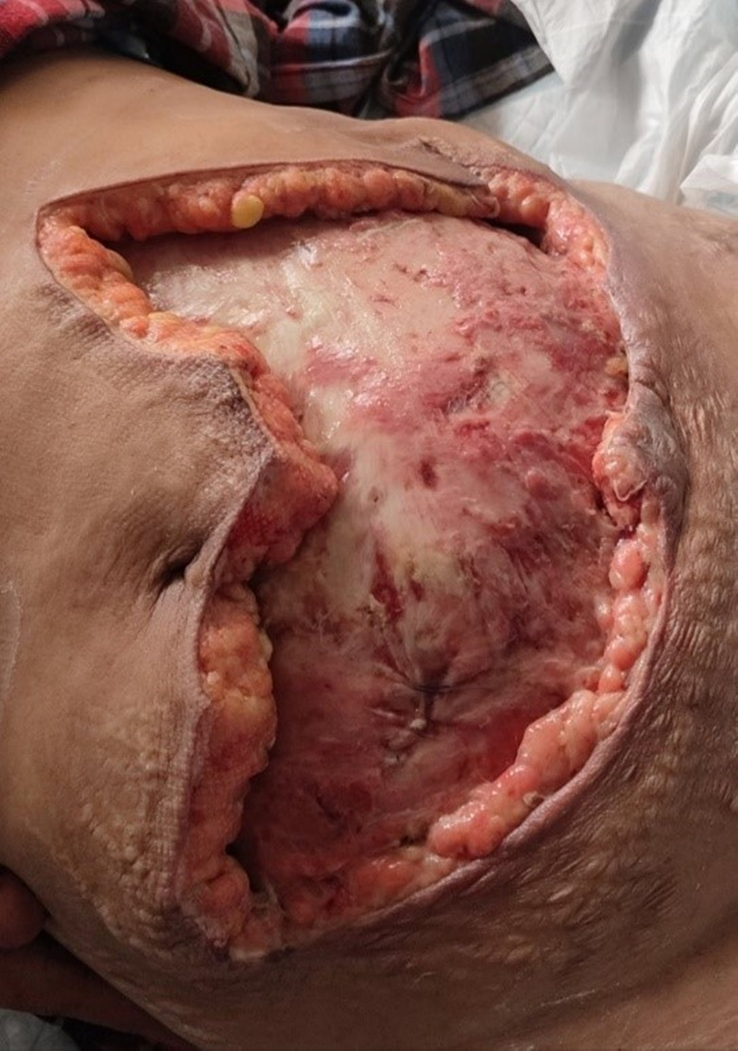
Healthy non-progressive wound prior to closure by abdominoplasty technique.

**Figure 8:**
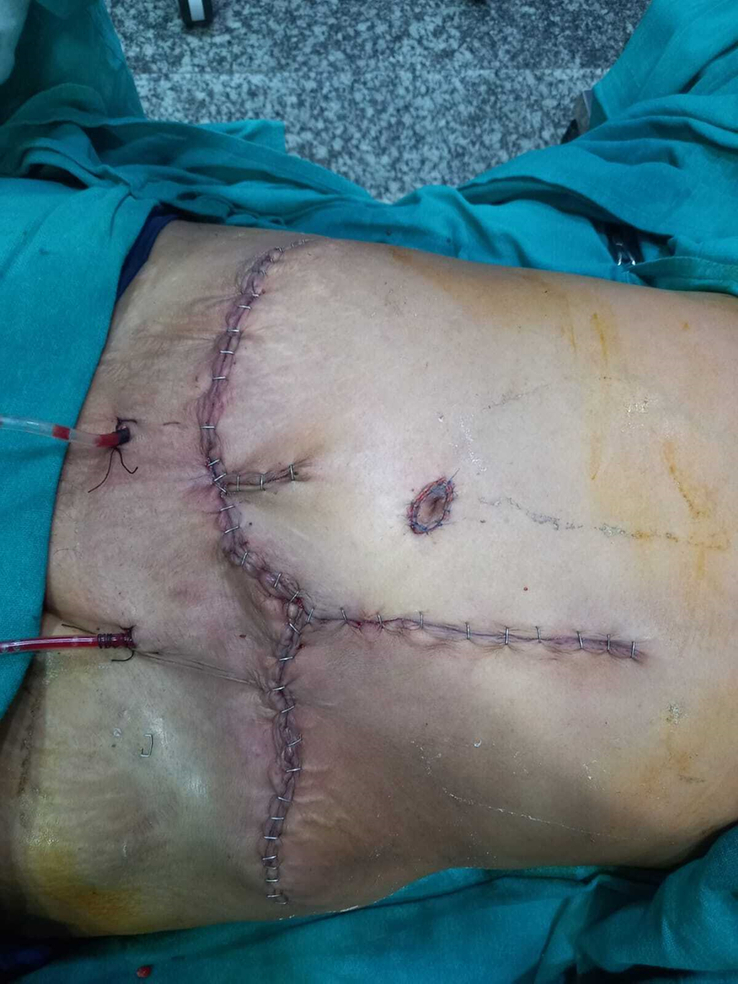
Wound closure with abdominoplasty concept.

On the fifth postoperative day, 900 ml of pleural fluid was tapped from the right side, and 250 ml of pleural fluid was tapped from the left side (Fig. 6). The wound started to show growth of granulation tissue (Fig. 7), and her symptoms improved. The patient was shifted to the ward on the same day after noticing clearance of the chest in comparison with prior films. Following evaluation of the wound on the seventh postoperative day, an abdominoplasty closure strategy was decided. On the eighth postoperative day, the patient underwent wound closure in an abdominoplasty fashion (Fig. 8). The postoperative period was uneventful, and the patient recovered well. Meleney’s gangrene appeared to require a skin graft; however, the concept of abdominoplasty resolved this case. The length of the hospital stay was also shortened as she was discharged post-drain removal on the 14th postoperative day. The patient made a full recovery over 1 month, and she was monitored.

## Discussion

Meleney’s gangrene is categorized under the umbrella term ‘necrotizing fasciitis.’ Necrotizing fasciitis is a life-threatening condition with a mortality rate of 34%^1^. Meleney’s gangrene is a rare form of necrotizing fasciitis that is commonly seen in immunocompromised states such as post-surgery, diabetes mellitus, old age, HIV, etc. It starts as a superficial skin infection or ulcer that spreads quickly to encompass the subcutaneous tissue, tiny vascular thrombosis leading to necrosis, extensive subcutaneous plane spreading, and later on gangrene^7^. Meleney’s gangrene is typically observed in the truncal region^8^.

Early detection of Meleney’s gangrene is usually challenging because its early presentation is similar to that of cellulitis or abscess. Our patient was also initially diagnosed and managed in line with cellulitis in another center before presenting to our institution. According to Frieschlag *et al*.^9^, the early detection and prompt management of Meleney gangrene within the first 24 h can lower mortality from 70% to 35%. At present, the suggestion was made to refer to all necrotizing infections as necrotizing soft tissue infections (NSTIs) and to follow the same methods for diagnosis and treatment planning. This will facilitate quicker identification and treatment, both of which are necessary to enhance the results and lower mortality in patients with NSTIs^4^.

The treatment of Meleney’s gangrene involves extensive surgical debridement and broad-spectrum antibiotics^1,9^. Surgical debridement and zinc oxide were available treatments when the illness was initially documented by Drs Brewer and Meleney in 1926^10^. Later, modern antibiotics replaced zinc oxide, aiding in the reduction of bacterial load, but the mainstay treatment was extensive surgical debridement and daily dressing. In our case, the patient underwent extensive debridement with removal of all necrotic tissues and administration of broad-spectrum antibiotics. Single debridement was proven to be sufficient as the wound showed no progression; thus, daily dressing was continued.

The usual approach is to continue dressing until the development of healthy granulation tissue and then cover the wound with the skin graft harvested from the thigh. However, as the soft tissue loss was infraumbilical, wound closure using the abdominoplasty technique was conceptualized. Abdominoplasty is a popular procedure for cosmetic purposes. Few cases of abdominoplasty for therapeutic purposes have been reported in the global literature^11^. Abdominoplasty, also known as tummy tuck, is a surgical procedure used to strengthen the muscles of the abdominal wall by removing extra skin and fat from the abdomen. It is frequently used by overweight people to decrease extra abdominal tissue after weight loss, either cosmetically or following bariatric surgery. However, for coverage of the defect following debridement of the infected and necrotic tissue, the same technique was used and was successful. This is the first documented case of extensive debridement of Meleney’s gangrene closed in an abdominoplasty fashion in Nepal.

## Conclusions

The case we report suggests an acquaintance with this rare condition among healthcare professionals for timely diagnosis and treatment, as time is the key determinant of the outcome of Meleney’s gangrene. We advocate the adoption of the advanced technique for prompt wound coverage, particularly in instances of Meleney’s gangrene, where the recovery period is protracted, leading to an extended hospital stay. Our approach employed the abdominoplasty technique traditionally utilized for cosmetic purposes. This strategic application resulted in a shortened hospital stay, totaling 14 days post extensive debridement.

## Ethical approval

Not available.

## Consent

Written informed consent was obtained from the patient for publication and any accompanying images. A copy of the written consent is available for review by the Editor-in-Chief of this journal on request.

## Sources of funding

Not available.

## Author contribution

S.H.: led data collection, concept of the study, and contributed to writing the case information; N.M.: literature review, revision, and editing of the manuscript in the final version; N.H.: literature review and revising and editing the manuscript. All authors were involved in manuscript drafting and revising and approved the final version.

## Conflicts of interest disclosure

The authors affirm that they are not related to or involved with any group or company that has a financial stake in the topic covered in this study.

## Research registration unique identifying number (UIN)

Not available.

## Guarantor

Mr Niran Maharjan, Department of Plastic Surgery, Sumeru City Hospital, Lalitpur, Nepal; e-mail: drnmaharjan@yahoo.com.

## Data availability statement

Not available.

## Provenance and peer review

Not commissioned, externally peer-reviewed.
